# Efficient Linkable Ring Signature Scheme over NTRU Lattice with Unconditional Anonymity

**DOI:** 10.1155/2022/8431874

**Published:** 2022-05-13

**Authors:** Qing Ye, Mengyao Wang, Hui Meng, Feifei Xia, Xixi Yan

**Affiliations:** School of Software, Henan Polytechnic University, Jiaozuo 454000, China

## Abstract

In cloud and edge computing, senders of data often want to be anonymous, while recipients of data always expect that the data come from a reliable sender and they are not redundant. Linkable ring signature (LRS) can not only protect the anonymity of the signer, but also detect whether two different signatures are signed by the same signer. Today, most lattice-based LRS schemes only satisfy computational anonymity. To the best of our knowledge, only the lattice-based LRS scheme proposed by Torres et al. can achieve unconditional anonymity. But the efficiency of signature generation and verification of the scheme is very low, and the signature length is also relatively long. With the preimage sampling, trapdoor generation, and rejection sampling algorithms, this study proposed an efficient LRS scheme with unconditional anonymity based on the e-NTRU problem under the random oracle model. We implemented our scheme and Torres et al.'s scheme, as well as other four efficient lattice-based LRS schemes. It is shown that under the same security level, compared with Torres et al.'s scheme, the signature generation time, signature verification time, and signature size of our scheme are reduced by about 94.52%, 97.18%, and 58.03%, respectively.

## 1. Introduction

In most scenarios involving data transmission, including blockchain, cloud computing, edge computing, etc., the sender of data usually wants to be anonymous, while the receiver of data always excepts the data to be reliable. Ring signature (RS) proposed by Rivest et al. [1] is a good technology that can meet the above requirements. RS has two essential security properties: (1) unforgeability, which requires the verifier is able to verify whether the signature was signed by a reliable signer; and (2) anonymity, which requires the verifier could not identify the real signer from a group of users. Similar to group signature [2, 3], RS is group-oriented. However, different from group signature, in RS, the group is formed spontaneously, that is, there is no special manager, and the setup and revocation procedures are not required. Any user can select a group of ring members and sign any message with his own private key and the public keys of other members without their consent. And the verifier only can verify whether the signature comes from a member in the ring without knowing which member the signer is.

Due to the anonymity of RS, it is widely used in anonymous tip off, e-cash [4], and other fields. It is worth noting that while protecting the anonymity of signers, RS also brings a new problem, that is, the same signer can sign multiple times without being detected.

In 2004, Liu et al. [5] introduced an extended property called linkability to RS, and the corresponding primitive is now known as linkable ring signatures (LRS). LRS not only satisfies the properties of ordinary RS (such as correctness, unforgeability, and anonymity) but also can be used to judge whether two different signatures are signed by the same signer (linkability). LRS is useful in situations where anonymity and nonrepeatability are required. For example, in the system of blockchain [6], if some user signs the same amount of money twice, LRS will help the verifier detect it and the verifier will deny the second signature, thus avoiding the so-called “double spending” problem. In smart grid systems [7], the electricity consumption data of users are automatically collected by the smart meter, and specific electricity consumption information is fed back to the service provider. Thus, malicious attackers can infer the life and rest rules of the user from the large amount of electricity consumption data recorded by the smart meter. LRS can not only conceal the specific information of the meter user but also eliminate the redundant data of the same meter and provide the system with abnormal user monitoring and tracking functions.

In 2013, Liu et al. [8] constructed an unconditional anonymous linkable ring signature (UALRS) scheme, which addressed the open problem that RS could not have linkability and strong anonymity simultaneously and made it more secure. RS schemes have two types of anonymity: computational anonymity and unconditional anonymity. Computational anonymity refers to the protection of anonymity under certain number theory problems. The anonymity of RS is destroyed if this potential problem can be solved by an adversary. By contrast, unconditional anonymity means that the probability that any adversary with unlimited computing power and time knows the actual signer of a given RS is no better than random guessing. In other words, assuming that there are *l* users in RS, the probability of any adversary with unlimited computing power and time correctly indicating the public key of the actual signer is no more than 1/*l*.

It is not difficult to design a RS scheme with unconditional anonymity. In fact, most traditional RS schemes can satisfy unconditional anonymity [1, 9–16]. However, it is not an easy work to construct a UALRS scheme. The difficulty lies in the following two aspects. First, in a computational anonymous linkable ring signature (CALRS) scheme, the linking tag can always be designed as a pseudorandom function about the private key of the signer based on some mathematical problem. But unconditional anonymity means that the adversary has unlimited computing power, that is it can calculate out the solution of any NP-hard problem, such as NTRU-SIS, large integer factorization, discrete logarithm, and the preimage of a given hash value. Therefore, only designing the linking tag using mathematical problems is not enough, and it should consider more skills. Second, in order to achieve unconditional anonymity, the generation and verification of a linking tag are often more complex, which may increase the length of public and private keys and signatures, as well as reduce the computational efficiency of the scheme. In fact, from 2004 to 2013, only the LRS scheme proposed by Liu et al. [8] can achieve unconditional anonymity.

The above schemes are all constructed based on classical number theory problems, that is, discrete logarithm and the decomposition of large integer problems. With the development of quantum computers, cryptosystems under classical number theory problems are faced with severe challenges. Shor [17] constructed a quantum algorithm in 1994 to solve the problem of large integer factorization in polynomial time under quantum computing conditions, and this algorithm made most existing public key cryptosystems no longer secure under quantum attacks.

In this case, post-quantum cryptography began to be studied by scholars in the field of cryptography. In the alternatives, lattice-based cryptography appeals to scholars because of its high efficiency, simplicity, high parallelizability, and strong provable security guarantees. In 2016, Libert et al. [18] constructed a lattice-based RS scheme based on zero-knowledge proofs and accumulators. Thereafter, other lattice-based RS schemes have been proposed [19–21]. In 2017, Yang et al. [22] proposed a lattice-based LRS scheme based on week pseudorandom functions, accumulators, and zero-knowledge proofs. In 2018, Baum et al. [23] proposed the lattice-based one-time LRS scheme based on the module-SIS problem (a variant of SIS problem) and module-LWE problem (a variant of LWE problem). In the same year, Alberto Torres et al. [24] proposed a lattice-based one-time LRS scheme based on the ring-SIS problem. Subsequently, Zhang et al. [25] proposed a LRS scheme over ideal lattice based on the homomorphic commitment scheme and ∑ protocol. In 2019, Liu et al. [26] proposed a lattice-based LRS scheme supporting stealth addresses under the module-SIS and module-LWE problems. In 2020, Beullens et al. [27] constructed a LRS scheme whose signature size scales logarithmically with the ring size from isogeny and lattice assumptions.

However, in the above lattice-based LRS schemes, only Alberto Torres et al.'s scheme [24] satisfies unconditional anonymity. By analyzing Torres et al.'s scheme, it is found that in order to achieve unconditional anonymity, the linking tag of Torres et al.'s scheme is generated using an *m*-dimensional polynomial vector over a polynomial ring. Since the linking tag is so large, Torres et al.'s scheme generates signatures *m* times longer than a normal CALRS scheme over a polynomial ring, and its efficiency in generating and verifying signatures is also significantly reduced.

Hoffstein et al. [28] proposed the NTRU lattice-based cryptosystem in 1996. Considering that it only involves multiplication on polynomial rings and small integer modulo operations, the NTRU-based cryptosystem usually requires smaller public and private keys and is more efficient compared with that on the general lattice. Therefore, it has received extensive attention from scholars. In 2016, Zhang et al. [29] proposed an efficient RS scheme on NTRU lattice whose security can be reduced to the e-NTRU problem (a variant of the SIS problem on NTRU lattice) in the random oracle model. In 2019, Lu et al. [30] constructed Raptor, a practical NTRU lattice-based LRS scheme based on a variant of chameleon hash functions. In 2021, Tang et al. [31] constructed an identity-based LRS scheme over NTRU lattice by employing the technologies of trapdoor generation and rejection sampling. The security of this scheme relies on the small integer solution (SIS) problem on NTRU lattice.

### 1.1. Our Contribution

**Figure 1:**
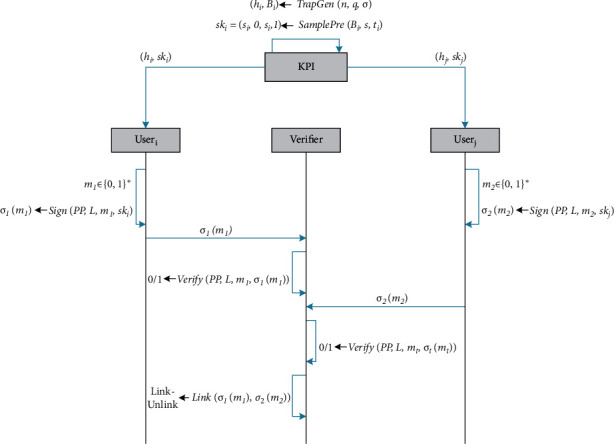
Linkable ring signature on NTRU lattice.

To reduce the signature size, as well as promote the efficiency of signature generation and verification of lattice-based UALRS scheme [24], in this study, a LRS scheme is reconstructed on NTRU lattice, and its architecture is shown in Figure 1. The main contributions of this article are as follows:

1. In the key generation stage, the public and private keys of the LRS scheme are generated by the trapdoor and the preimage sampling algorithms on NTRU lattice. Then, the linking tag is produced by the public and private keys of the signer, and a LRS is generated based on the signature algorithm of Zhang et al. [29] combined with the rejection sampling algorithm.
2. In terms of security analysis, strict security proof is conducted based on the security model of UALRS proposed by Liu et al. [8]. The result of the proof shows that the unforgeability and linkability of the proposed scheme can be reduced to the difficulty of e-NTRU problem under the random oracle model, and, meanwhile, the proposed scheme satisfies unconditional anonymity.
3. In terms of performance analysis, the proposed scheme is compared with the latest and efficient lattice-based LRS schemes in [23, 24, 26, 27, 30], and a detailed analysis is given. The possible parameter settings of the proposed scheme are also analyzed and provided under the premise of ensuring the security of the proposed scheme.
4. We implement our scheme and Torres et al.'s scheme [24], as well as other four efficient lattice-based LRS schemes [23, 26, 27, 30], and it is shown that under the same security level, the signature generation and verification time of the proposed scheme are respectively reduced by 56.61% and 65.18%. Especially compared with Torres et al.'s scheme, the signature generation and verification time of the proposed scheme are respectively reduced by 94.52% and 97.18%, and the signature size of the proposed scheme is reduced by 58.03% on average.

### 1.2. Paper Organization

In Section 2, we introduce some definitions, lemmas, difficult problems, and related algorithms which we will use to construct the scheme. We introduce the definition of LRS and the relevant security model in Section 3.  Section 4 contains the construction and correctness statement of the LRS scheme and the proof of correctness.  Section 5 contains the security statements of the proposed scheme and the proofs of unforgeability, unconditional anonymity, and linkability. In Section 6, we discuss the parameter settings and post-quantum security of the proposed scheme. Finally, in Section 7 and Section 8, we respectively give the performance analysis and experimental results of the proposed scheme and the lattice-based LRS schemes of [23, 24, 26, 27, 30] and also make a comparison between them.

## 2. Preliminaries

### 2.1. Symbol Definition

Descriptions of the used notations are listed in Table 1.

### 2.2. Related Definitions of NTRU Lattice

**Definition 1 (lattice).**

Lattice Λ generated by *m* linearly independent vectors **b**_1_, **b**_2_, ⋯, **b**_*m*_ ∈ *ℝ*^*n*^ is the set of linear combinations of all integer coefficients of the *m* linearly independent vectors, namely

(1) $$\begin{array}{c} \Lambda =L\left(b_{1},b_{2},\cdots ,b_{m}\right)=\left\{\sum_{i=1}^{m}a_{i}b_{i}|a_{i}\in \mathbb{Z}\right\}, \end{array}$$

where *m* and *n* are the rank and dimension of lattice Λ, respectively, and **b**_1_, **b**_2_, ⋯, **b**_*m*_ is called a basis of lattice Λ.

**Definition 2 (convolutional polynomial ring).**

Let *R*=*ℤ*[*x*]/(*x*^*n*^+1) be an ordinary polynomial ring. If the addition operation remains unchanged and the multiplication operation is replaced by a convolution operation on *R*, then *R* is called a convolution polynomial ring. Similarly, given a prime number *q*, the modulus convolution polynomial ring is *R*_*q*_=*R*/*qR*.

Let *f*=∑_*i*=0_^*n*−1^*f*_*i*_*x*^*i*^, *g*=∑_*i*=0_^*n*−1^*g*_*i*_*x*^*i*^ ∈ *R*_*q*_, then the two operations on *R*_*q*_ are defined as follows:

(i) Addition operation +:
  (2) $$\begin{array}{c} f+g=\overset{n-1}{\underset{i=0}{\sum }}\left(f_{i}+g_{i}\right)x^{i}\mathrm{mod}q\in R_{q}. \end{array}$$
(ii) Convolution operation *∗*:
  (3) $$\begin{array}{c} f*g=f\cdot g\mathrm{mod}\left(x^{n}+1\right)\mathrm{mod}q\in R_{q}. \end{array}$$

**Definition 3 (anticirculant matrix).**

Let the coefficient vector of polynomial *f* be (*f*_0_, *f*_1_, ⋯, *f*_*n*−1_). Then, the coefficient vector of polynomial *x* · *f* is (−*f*_*n*−1_, *f*_0_, ⋯, *f*_*n*−2_) and the coefficient vector of polynomial *x*^*n*−1^ · *f* is (−*f*_1_, −*f*_2_, ⋯, *f*_0_). The anti-circulant matrix defined by polynomial *f* is as follows:

(4) $$\begin{array}{c} A_{n}\left(f\right)=\left[\begin{array}{c} \;f_{0}\\ -f_{n-1}\\ \vdots \\ -f_{1} \end{array}\right)\;\left(\begin{array}{c} \;f_{1}\;\cdots \;f_{n-1}\;\\ \;f_{0}\;\cdots \;f_{n-2}\\ \;\vdots \cdots \;\vdots \\ -f_{2}\;\cdots \;f_{0} \end{array}\right]=\left[\begin{array}{c} f\\ x\cdot f\\ \vdots \\ x^{n-1}\cdot f \end{array}\right]. \end{array}$$

**Definition 4 .**

(NTRU lattice). Let a positive integer *q* ≥ 2, *n* is a power of two and *f*, *g* ∈ *R*_*q*_, *f*^−1^ ∈ *R*_*q*_ be the inverse of *f*, *h*=*g∗f*^−1^mod*q*. The NTRU lattice corresponding to *q* and *h* is as follows:

(5) $$\begin{array}{c} \Lambda_{q,h}=\left\{\left(u,v\right)\in R^{2}|u+v*h=0\mathrm{mod}q\right\}. \end{array}$$

Apparently, lattice Λ_*q*,*h*_ is a 2*n*-dimensional full-rank lattice, and $A_{q,h}=\left(\begin{array}{c} -A_{n}\left(h\right)I_{n}\\ \;qI_{n}O_{n} \end{array}\right)\in {\mathbb{Z}}_{q}^{2n\times 2n}$ is a set of basis matrices. **A**_*q*,*h*_ can be uniquely determined by the polynomial *h* ∈ *R*_*q*_, whereas the others can be compressed during storage. Thus, the storage space required is relatively small. However, in NTRU lattice-based cryptographic schemes, **A**_*q*,**h**_ cannot be used as a trapdoor basis because it has poor orthogonality.

**Definition 5 .**

(discrete gaussian distribution) [32]. For any *σ* > 0 and *m*-dimensional integer lattice Λ, the discrete Gaussian distribution on integer lattice Λ with vector **c** ∈ *ℝ*^*m*^ as the center and *σ* as the parameter is defined as follows:

(6) $$\begin{array}{c} \forall x\in \Lambda ,D_{\Lambda ,c,\sigma }^{m}\left(x\right)=\frac{\rho_{c,\sigma }^{m}\left(x\right)}{\rho_{c,\sigma }^{m}\left(\Lambda \right)}, \end{array}$$

where *ρ*_**c**,*σ*_^*m*^(**x**)=exp(−*π*‖**x** − **c**^2^‖/*σ*^2^). When *c*=0, let *ρ*_**c**,*σ*_^*m*^ and *D*_Λ,**c**,*σ*_^*m*^ be abbreviated as *ρ*_*σ*_^*m*^ and *D*_Λ,*σ*_^*m*^, respectively. And throughout the article, *D*_**c**,*σ*_^*m*^ denotes the discrete Gaussian distribution over *ℤ*^*m*^.

### 2.3. Hardness Assumption

**Definition 6 (NTRU small-integer solution, NTRU-SIS) [33].**

For a polynomial *h*=*g∗f*^−1^mod*q* ∈ *R*_*q*_ and a real number *β* > 0, to find two nonzero polynomials (*u*, *v*) ∈ *R*_*q*_^2^ such that *u*+*v∗h*=0mod*q* and ‖*u*‖, ‖*v*‖ ≤ *β*.

**Definition 7 (extended NTRU, e-NTRU) [29].**

Given *N* polynomials *h*_*i*_=*g*_*i*_*∗f*_*i*_^−1^mod*q* ∈ *R*_*q*_, *i* ∈ {1, ⋯, *N*}, where *N* ≪ *q*, to find a tuple of short polynomials (*u*_*i*_, *v*_*i*_) ∈ *R*_*q*_^2^, *u*_*i*_, *v*_*i*_ ≠ 0mod*q*, *i* ∈ {1, ⋯, *N*} such that

(7) $$\begin{array}{c} \sum_{i=1}^{N}\left(u_{i}+v_{i}*h_{i}\right)=0\mathrm{mod}q,\left\|u_{i}\right\|,\left\|v_{i}\right\|\leq \beta ,i\in \left\{1,\cdots ,N\right\}. \end{array}$$

**Theorem 1 (see [29]).**

Let integer *k* > 0, *n*=2^*k*^, *q*=1mod2*n* and integer *N* ≪ *q*, then the e-NTRU problem is polynomially equivalent to the NTRU-SIS problem.

### 2.4. Related Algorithm

**Lemma 1 (see [34]).**

Let an integer *n*=2^*k*^ for *k* > 0, a prime number *q*=1mod2*n*, and a parameter $\sigma =1.17\sqrt{q/2n}$. Then, a probabilistic polynomial time (PPT) algorithm TrapGen(*n*,*q*,*σ*) can output a sample matrix **B**_*f*,*g*_ ∈ *ℤ*_*q*_^2*n*×2*n*^ from (a distribution close to) *D*_*h*,*σ*_^2*n*×2*n*^ and a polynomial *h*=*g∗f*^−1^mod*q* ∈ *R*_*q*_ on the NTRU lattice Λ_*h*,*q*_.

**Lemma 2 (see [34]).**

Given a matrix **B**_*f*,*g*_ and a parameter $s=0.585/\pi \sqrt{q\;\ln\left(2+2/\eta \right)}$ for *η*=2^−*λ*^/2*n*, where *λ* is the security parameter. For any polynomial *t* ∈ *R*_*q*_, a PPT algorithm *SamplePre*(**B**_*f*,*g*_, *s*, *t*) may output **z**=(*z*_1_, *z*_2_) ← *D*_*h*^⊥^+**c**,*s*_, such that $z_{1}+z_{2}*h=t,\left\|z\right\|\leq s\sqrt{2n}$.

**Definition 8 (rejection sampling algorithm) [35].**

In 2012, Lyubashevsky proposed rejection sampling technique for the first time and gave the first signature scheme without trapdoor on lattice with this technique. It can be applied to the signature system and can make the distributions of the signature and private key independent of each other. Thus, it can effectively prevent the leakage of the private key.

**Lemma 3 .**

Let *V*={**v** ∈ *ℤ*^*m*^: ‖**v**‖ < *t*}, $\sigma =\omega \left(t\sqrt{\log m}\right)$, and *h* : *V*⟶*ℝ* is a probability distribution. Then, for constant *M*=*O*(1), the statistical distance of output distributions of Algorithms 1 and 2 is less than 2^−*ω*(log*m*)^/*M*.

**Algorithm 1 .**

*v* ← *h*, **z** ← *D*_**v**,*σ*_^*m*^, output (**z**, *v*) with probability min(*D*_*σ*_^*m*^(**z**)/*MD*_**v**,*σ*_^*m*^(**z**),1).

**Algorithm 2 .**

*v* ← *h*, **z** ← *D*_*σ*_^*m*^, output (**z**, *v*) with probability 1/*M*.

Furthermore, the output probability of Algorithm 1 is at least 1 − 2^−*ω*(log*m*)^/*M*.

## 3. Security Model

In this section, we present our security model and define related security concepts.

### 3.1. LRS Definition

A LRS scheme consists of the following five PPT algorithms:

1. Setup(1^*λ*^): On input a security parameter *λ*, it outputs system public parameters *PP*.
2. KeyGen(*PP*): On input the public parameters *PP*, it outputs a public/private key pair (*pk*_*i*_, *sk*_*i*_).We denote by *SK* and *PK* the domains of possible private and public keys, respectively.
3. Sign(*PP*, *L*, *m*, *sk*_*k*_): On input the public parameters *PP*, a public key list *L*, a message *m* ∈ {0,1}^*∗*^, and private key *sk*_*k*_, it outputs a signature *σ*(*m*), which contains a linking tag *I*.
4. Verify(*PP*, *L*, *m*, *σ*(*m*)): On input the system public parameters *PP*, a public key list *L*, a message *m* ∈ {0,1}^*∗*^, and a signature *σ*(*m*), if *σ*(*m*) is valid, it outputs “1”; otherwise, it outputs “0.”
5. Link(*σ*(*m*_1_), *σ*(*m*_2_)): On input two signatures *σ*_1_(*m*_1_), *σ*_2_(*m*_2_), where *σ*_1_(*m*_1_) and *σ*_2_(*m*_2_) are the signatures of different messages *m*_1_ and *m*_2_ under the same ring, which contain linking tags *I*_1_ and *I*_2_, respectively. It checks whether $I_{1}\overset{?}{=}I_{2}$ and outputs “Link” if *I*_1_=*I*_2_; otherwise, it outputs “Unlink.” “Link” means that the two signatures are generated by the same signer, and “Unlink” means that the two signatures are generated by different signers.

**Definition 9 (correctness).**

Correctness for LRS contains verification correctness and linking correctness simultaneously.

(i) *Verification Correctness*: For a valid signature *σ*(*m*), the probability of the algorithm Verify(*PP*, *L*, *m*, *σ*(*m*)) outputting “0” is negligible.
(ii) *Linking Correctness:* For two valid signatures *σ*_1_(*m*_1_), *σ*_2_(*m*_2_) generated by using the same private key, the probability of the algorithm Link(*σ*(*m*_1_), *σ*(*m*_2_)) outputting “Unlink” is negligible. The formal definition of the correctness of the LRS scheme is shown in the following expressions:
  (8) $$\begin{array}{c} \mathrm{Pr}\left[{}^{\prime \prime }0^{\prime \prime }\leftarrow \text{Verify}\left(PP,L,m,\sigma \left(m\right)\right)\left|\begin{array}{c} \;PP\leftarrow \text{Setup}\left(1^{\lambda }\right)\\ \left(pk,sk\right)\leftarrow \text{KeyGen}\left(PP\right)\\ \sigma \left(m\right)\leftarrow \text{Sign}\left(PP,L,m\text{,}sk\right) \end{array}\right.\right]\leq negl\left(\lambda \right). \end{array}$$
  (9) $$\begin{array}{c} \mathrm{Pr}\left[\text{“Unlink“}\leftarrow \text{Link}\left(\sigma \left(m_{1}\right),\sigma \left(m_{2}\right)\right)\left|\begin{array}{c} PP\leftarrow \text{Setup}\left(1^{\lambda }\right)\\ \left(pk,sk\right)\leftarrow \text{KeyGen}\left(PP\right)\\ \sigma \left(m_{1}\right)\leftarrow \text{Sign}\left(PP,L_{1},m_{1},sk\right)\\ \sigma \left(m_{2}\right)\leftarrow \text{Sign}\left(PP,L_{2},m_{2},sk\right) \end{array}\right.\right]\leq negl\left(\lambda \right). \end{array}$$

### 3.2. Security Model

Generally, a LRS scheme should satisfy three security properties, namely unforgeability, anonymity, and linkability. According to the security model of UALRS proposed by Liu et al. [8] in 2013, this study uses a series of games between an adversary *A* and a challenger *S* to describe the security model of LRS. Supposing there are *l* members in the ring, these three properties are described as follows:

Before defining unforgeability, anonymity, and linkability, we consider the following oracles, which together simulate the adversary's ability to break the security of the scheme.

 
*JO* (*Joining Oracle*): *A* inputs member index *k*, and *S* outputs the corresponding public key *pk*_*k*_ ∈ *PK* to *A*
 
*CO* (*Corruption Oracle*): *A* inputs a public key *pk*_*k*_ ∈ *PK*, which is a query output of *JO*, and *S* returns the corresponding private key *sk*_*k*_ ∈ *SK*
 
*SO* (*Signing Oracle*): *A* inputs a public key list *L*={*pk*_*i*_}_1≤*i*≤*l*_ ∈ *PK*, and a message *m* ∈ {0,1}^*∗*^, and *S* returns a valid signature *σ*(*m*)

In addition, in the random oracle model, a random oracle model *HO* is provided for users to query.

#### 3.2.1. Unforgeability

It means that users outside the ring cannot successfully forge a legal signature under the ring. That is, if there is no private key of a member in the ring, even if the adversary obtains multiple valid message signature pairs, the probability of the adversary forging a valid signature successfully is negligible. Unforgeability for the LRS scheme is defined by the following game between an adversary *A* and a challenger *S*, in which *A* is given access to oracles *JO*, *CO*, *SO*, and *HO*:

- The system public parameters *PP* are generated by challenger *S* and given to *A*
- *A* can access the oracles adaptively
- *A* gives *S* a list *L*={*pk*_*i*_}_1≤*i*≤*l*_ of public keys, a message *m* ∈ {0,1}^*∗*^, and a signature *σ*(*m*)

*A* wins the game if

- Verify(*PP*, *L*, *m*, *σ*(*m*))=^″^1^″^
- All public keys in *L* are obtained by querying *JO*
- Any public key in *L* has not been input to *CO*
- *σ*(*m*) is not obtained by querying *SO*

We express it as

(10) $$\begin{array}{c} \mathrm{Ad}v_{A}^{\mathrm{Unf}}=\mathrm{Pr}\left[A\text{wins the game}\right]. \end{array}$$

**Definition 10 (unforgeability).**

If the advantage *Adv*_*A*_^*Unf*^ of any PPT adversary *A* to win the unforgeability game is negligible, then the LRS scheme is unforgeable.

#### 3.2.2. Unconditional Anonymity

It means that given a ring signature, no one can guess the real signer. In other words, given the public keys of all the members of the ring, it is impossible for anyone to tell the public key of the actual signer with a probability larger than 1/*l*, where *l* denotes the cardinality of the ring, even the adversary has unlimited computing time and resources. The unconditional anonymity of LRS is described by the following game between an adversary *A* and a challenger *S*, where *A* is granted access to oracle *JO*:

- The system public parameters *PP* are generated by challenger *S* and given to *A*;
- *A* can access the oracle *JO* adaptively;
- *A* gives *S* a public key list *L*={*pk*_*i*_}_1≤*i*≤*l*_, which are query outputs of *JO*, and a message *m*^*∗*^ ∈ {0,1}^*∗*^. *S* randomly samples *b* ∈ {1, ⋯, *l*}, uses the signature key *sk*_*b*_ corresponding to *pk*_*b*_ to run algorithm Sign(*PP*, *L*, *m*, *sk*_*b*_), and generates and gives *A* the signature *σ*(*m*^*∗*^); and
- *A* returns the guess value *b*′∈{1, ⋯, *l*}.

We express it as

(11) $$\begin{array}{c} \mathrm{Ad}v_{A}^{\mathrm{Anon}}=\left|\mathrm{Pr}\left[b^{\prime }=b\right]-1/l\right|. \end{array}$$

**Definition 11 (unconditional anonymity).**

If the advantage *Adv*_*A*_^*Anon*^ of any unbounded adversary *A* to win the anonymity game is negligible, then the LRS scheme is called to be unconditional anonymous.

It is worth noting that though only *JO* is given to *A*, since *A* has unbounded computation power, it can calculate out the solution of any NP-hard problem, such as NTRU-SIS, large integer factorization, discrete logarithm, as well as the preimage of a given hash value. Therefore, unconditional anonymity in fact requires that in this case, *A* is still unable to reveal the pubic key of the actual signer of a RS with a probability higher than 1/*l*.

#### 3.2.3. Linkability

It means that two signatures generated by the same ring member can be linked. That is, an adversary who has less than two members' private keys in the ring cannot generate two valid signatures determined by the linking algorithm as “Unlink.” The linkability of a LRS scheme is described by the following game between an adversary *A* and a challenger *S*, where *A* is granted access to oracles *JO*, *CO*, *SO*, and *HO*:

- The system public parameters *PP* are generated by challenger *S* and given to *A*
- *A* can access the oracles adaptively
- *A* gives *S* two sets *L*_1_={*pk*_*i*_}_1≤*i*≤*l*_1__ and *L*_2_={*pk*_*i*_}_1≤*i*≤*l*_2__, messages *m*_1_, *m*_2_ ∈ {0,1}^*∗*^, and signatures *σ*(*m*_1_) and *σ*(*m*_2_), where *σ*(*m*_1_) and *σ*(*m*_2_) contain the corresponding linking tags *I*_1_, *I*_2_, respectively

*A* wins the game if

- All public keys in *L*_1_ ∪ *L*_2_ are query outputs of *JO*
- For *i*=1,2, *Verify*(*PP*, *L*_*i*_, *m*_*i*_, *σ*(*m*_*i*_))=″1″ such that *σ*(*m*_*i*_) is not an output of *SO*
- *CO* has been queried less than two times
- *Link*(*σ*(*m*_1_), *σ*(*m*_2_))=“Unlink“

We express it as

(12) $$\begin{array}{c} \mathrm{Ad}v_{A}^{\mathrm{Link}}=\mathrm{Pr}\left[A\text{wins the game}\right]. \end{array}$$

**Definition 12 (linkability).**

If the advantage *Adv*_*A*_^*Link*^ of any PPT adversary *A* to win the linkability game is negligible, then the LRS scheme is linkable.

## 4. Scheme Construction

(1) Setup(1^*λ*^, 1^*n*^): On input the security parameter *λ* and integer *n*=2^*k*^, where *k* > 0, a ring of *l*=*ω*(log*n*), a prime *q*=1mod2*n*, two parameters $\sigma =1.17\sqrt{q/2n}$ and $s=0.585/\pi \sqrt{q\;\ln\left(2+2/\eta \right)}$, where *η*=2^−*λ*^/2*n*, choose a collision-resistant hash function *H* : {0,1}^*∗*^⟶{0,1}^*n*^, and output *PP*=(*q*, *σ*, *s*, *H*).
(2) *KeyGen*(*PP*): On input the system public parameters *PP*, the following steps should be performed:
  (i) Run the trapdoor generation algorithm TrapGen(*n*,*q*,*σ*) to generate {*h*_*i*_ ∈ *R*_*q*_, **B**_*i*_ ∈ *ℤ*_*q*_^2*n*×2*n*^};
  (ii) Randomly choose *t*_*i*_ ∈ *R*_*q*_, and let *sk*_*i*_=(*s*_*i*,0_, *s*_*i*,1_)=SamplePre(**B**_*i*_, *s*, *t*_*i*_) such that $s_{i,0}+s_{i,1}*h_{i}=t_{i},\left\|\left(s_{i,0},s_{i,1}\right)\right\|\leq s\sqrt{2n}$; and
  (iii) Output a public key list *L*={*h*_*i*_}_1≤*i*≤*l*_, and the private key for the member *i*: *sk*_*i*_=(*s*_*i*,0_, *s*_*i*,1_).
(3) Sign(*PP*, *L*, *m*, *sk*_*k*_): On input the system public parameters *PP*, the public key list *L*={*h*_*i*_}_1≤*i*≤*l*_, a message *m* ∈ {0,1}^*∗*^, and a private key *sk*_*k*_=(*s*_*k*,0_, *s*_*k*,1_), the member *k* performs the following steps:
  (i) Compute linking tag
    (13) $$\begin{array}{c} I=s_{k,0}+s_{k,1}*h_{k}. \end{array}$$
  (ii) For 1 ≤ *i* ≤ *l*, sample random vectors **r**_*i*,0_, **r**_*i*,1_ ← *D*_*s*_^*n*^.
  (iii) Let
    (14) $$\begin{array}{c} v=\;H\left(\sum_{1\leq i\leq l}\left(r_{i,0}+r_{i,1}*h_{i}\right),L,m,I\right). \end{array}$$
  (iv) If *i* ≠ *k*, compute
    (15) $$\begin{array}{c} z_{i}=\left(z_{i,0},z_{i,1}\right)=\left(r_{i,0},r_{i,1}\right). \end{array}$$
  if *i*=*k*, compute
    (16) $$\begin{array}{c} z_{k}=\left(z_{k,0},z_{k,1}\right)=\left(s_{k,0}*v+r_{k,0},s_{k,1}*v+r_{k,1}\right). \end{array}$$
  (v) Continue with probability min(*D*_*s*_^*n*^(*z*_*k*_)/*MD*_*s∗v*,*s*_^*n*^(*z*_*k*_), 1), where *M*=*O*(1); otherwise restart.
  (vi) Output signature *σ*(*m*)=(*m*, (**z**_*i*_)_1≤*i*≤*l*_, **v**, *I*).
(4) Verify(*PP*, *L*, *m*, *σ*(*m*)): On input the system parameters *PP*, the public key list *L*={*h*_*i*_}_1≤*i*≤*l*_, a message *m* ∈ {0,1}^*∗*^, and a signature *σ*(*m*)=(*m*, (**z**_*i*_)_1≤*i*≤*l*_, **v**, *I*), output “1” if and only if the following conditions are true; otherwise, output “0”:
  (17) $$\begin{array}{c} \left(\text{i}\right)v=H\left(\sum_{1\leq i\leq l}\left(z_{i,0}+z_{i,1}*h_{i}\right)-Iv,L,m,I\right). \end{array}$$
  (18) $$\begin{array}{c} \left(\text{ii}\right)\text{For }1\leq i\leq l,\text{0}\leq \left\|\left(z_{i,0},z_{i,1}\right)\right\|\leq s\sqrt{2n}. \end{array}$$
(5) Link(*σ*(*m*_1_), *σ*(*m*_2_)): On input two signatures *σ*(*m*_1_) and *σ*(*m*_2_), which contains linking tags *I*_1_ and *I*_2_, respectively, the following steps should be performed:

Verify whether $I_{1}\overset{?}{=}I_{2}$. If *I*_1_=*I*_2_, then return “Link”; otherwise, return “Unlink.”

**Theorem 2 (correctness).**

The proposed LRS scheme satisfies correctness.

**Proof**

Assuming *σ*(*m*)=(*m*, (**z**_*i*_)_1≤*i*≤*l*_, **v**,*I*) is a signature generated by a member of the ring according to the algorithms under public key set *L*={*h*_*i*_}_1≤*i*≤*l*_, then the following equation holds:

(19) ∑1≤i≤lzi,0+zi,1∗hi−Iv=zk,0+z∗k,1 hk−Iv+∑1≤i≤l,i≠kzi,0+zi,1∗hi=sk,0+s∗k,1 hkv+rk,0+rk,1∗hk−Iv+∑1≤i≤l,i≠kri,0+ri,1∗hi.

Given that *s*_*k*,0_+*s*_*k*, 1_^ ^*∗h*_*k*_=*I*, we have

(20) $$\begin{array}{c} \sum_{1\leq i\leq l}\left(z_{i,0}+z_{i,1}*h_{i}\right)-Iv=\sum_{1\leq i\leq l}\left(r_{i,0}+r_{i,1}*h_{i}\right). \end{array}$$

Hence,

(21) $$\begin{array}{c} v=H\left(\sum_{1\leq i\leq l}\left(z_{i,0}+z_{i,1}*h_{i}\right)-Iv,L,m,I\right). \end{array}$$

By using the rejection sampling algorithm described in Definition 8, the distribution of (**z**_*i*,0_, **z**_*i*,1_) is close to *D*_*s*_^*n*^(**z**_*i*_) for 1 ≤ *i* ≤ *l*. Thus, by Lemma 3, we have **z**_**i**_=(**z**_*i*,0_, **z**_*i*,1_) satisfies $\left\|z_{i}\right\|\leq s\sqrt{2n}$ with a probability at least 1 − 2^−*ω*(log*n*)^. Therefore, the proposed scheme satisfies verification correctness.

Assume member *k* calculates the linking tags of messages *m*_1_ and *m*_2_ as *I*_1_ and *I*_2_, respectively. In the proposed scheme, *I*_1_=*s*_*k*,0_+*s*_*k*,1_*∗h*_*k*_ and *I*_2_=*s*_*k*,0_+*s*_*k*,1_*∗h*_*k*_ are generated by the signer's public and private keys, and thus this scheme satisfies linking correctness. This completes the proof.

## 5. Security Analysis

**Theorem 3 (unforgeability).**

Under the random oracle model, when the e-NTRU problem is intractable, the proposed LRS scheme is unforgeable.

**Proof**

*Setup Phase*: To solve the e-NTRU problem, *S* gets an instance (*h*_*i*_)_1≤*i*≤*l*_

*Query Phase*: Adversary *A* is allowed to access oracles *JO*, *CO*, *SO*, and *HO*, and *S* responds as follows:

(i) *H*: *A* inputs ((**r**_*i*,0_, **r**_*i*,1_)_1≤*i*≤*l*_, *L*, *m*, *I*, *k*), *S* first checks whether there is the relevant record in the list *list*_*H*_. If so, then the same query result is returned to *A.* Otherwise, *S* randomly picks and gives *A* an integer **v**, and adds the tuple ((**r**_*i*,0_, **r**_*i*,1_)_1≤*i*≤*l*_, *L*, *m*, *I*, **v**, *k*) to the list *list*_*H*_.
(ii) *JO*: Suppose *A* can only access the oracle *JOl*′ times at most, where *l*′ ≥ *l*. *S* selects a subset *X*_*l*_ with *l* random indexes. *S* assigns (*h*_*i*_)_1≤*i*≤*l*_ to these *l* indexes as their public keys, respectively. Moreover, for these *l* indexes, *S* does not know the corresponding private keys. We use *l*+1, ···, *l*′ to denote other indexes. With regard to other *l*′ − *l* indexes, *S* obtains the public and private keys according to the algorithm KeyGen(*PP*). *A* inputs index *j* to query, and *S* outputs the corresponding public key.
(iii) *CO*: *A* inputs a public key *pk*_*i*_=*h*_*i*_, *S* checks whether *i* belongs to *X*_*l*_. If so, then *S* stops; otherwise, *S* outputs the corresponding private key.
(iv) *SO*: *A* inputs a ring public key set *L*={*h*_*i*_}_1≤*i*≤*l*_, a public key *h*_*k*_, where *k* ∈ {1, ⋯, *l*}, and a message *m* ∈ {0,1}^*∗*^. *S* performs as follows:
  1. If *h*_*k*_ does not correspond to any element in the subset *X*_*l*_, then *S* knows its private key and generates the signature according to the signature algorithm Sign(*PP*, *L*, *m*, *sk*_*k*_). Otherwise, we assume that *h*_*k*_ is obtained by *JO*.
  2. *S* checks the list *list*_*H*_ to find the record ((**r**_*i*,0_, **r**_*i*,1_)_1≤*i*≤*l*_, *L*, *m*, *I*, **v**, *k*) corresponding to the index *k*. Then, *S* randomly chooses **z**_*i*,0_, **z**_*i*,1_ ← *D*_*s*_^*n*^ and sets the output of *H*(∑_1≤*i*≤*l*_(**z**_*i*,0_+**z**_*i*,1_*∗h*_*i*_) − *I ***v**, *L*, *m*, *I*) to **v**.
  3. *S* returns a signature *σ*(*m*)=(*m*, (**z**_*i*_)_1≤*i*≤*l*_, **v**, *I*) with probability min(*D*_*s*_^*n*^(*z*_*k*_)/*MD*_*s*_*k*_*∗v*,*s*_^*n*^(*z*_*k*_), 1), where *M*=*O*(1).

*Forgery Phase:* After the simulation, *A* gives signature *σ*(*m*^*∗*^)=(*m*^*∗*^, (**z**_*i*,0_^*∗*^, **z**_*i*,1_^*∗*^)_1≤*i*≤*l*_, **v**^*∗*^, *I*^*∗*^) about {*PP*, *m*^*∗*^, *L*}^*∗*^ to *S* satisfying the following conditions:

- Verify(*PP*, *L*^*∗*^, *m*^*∗*^, *σ*(*m*^*∗*^))=^″^1^″^
- All of the public keys *pk*_*i*_=*h*_*i*_ in *L*^*∗*^ are query outputs of *JO*
- *A* did not query *CO* about the public keys in *L*^*∗*^
- *σ*(*m*^*∗*^) is not a query output of *SO*

*Analysis*. Assuming the signature *σ*(*m*^*∗*^) is a valid signature, the following shows how *S* can solve the e-NTRU problem using the forged results of *A*. We will consider the following two situations:

(i) If **v**^*∗*^ appears in the *SO*, and assume that *σ*(*m*)=(*m*, (*z*_*i*,0_, *z*_*i*,1_)_1≤*i*≤*l*_, *v*^*∗*^, *I*) is a query output of *SO*. Given that the signature is valid, it satisfies
  (22) $$\begin{array}{c} v^{*}=H\left(\sum_{1\leq i\leq l}\left(z_{i,0}+z_{i,1}*h_{i}\right)-Iv^{*},L,m,I\right). \end{array}$$
Given that *A* successfully forged the signature, there is
  (23) $$\begin{array}{c} v^{*}=H\left(\sum_{1\leq i\leq l}\left(z_{i,0}^{*}+z_{i,1}^{*}*h_{i}\right)-I^{*}v^{*},L^{*},m^{*},I^{*}\right). \end{array}$$
When the function *H* collides, *S* aborts (Abort I). Otherwise, from (22) and (23), there is
  (24) $$\begin{array}{c} L^{*}=L,m^{*}=m,I^{*}=I, \end{array}$$
  (25) $$\begin{array}{c} \sum_{1\leq i\leq l}\left(z_{i,0}-z_{i,0}^{*}\right)+\left(z_{i,1}-z_{i,1}^{*}\right)*h_{i}=0\mathrm{mod}q. \end{array}$$
Therefore, [(**z**_*i*,0_^*∗*^ − **z**_*i*,0_),(**z**_*i*,0_^*∗*^ − **z**_*i*,1_)]_1≤*i*≤*l*_ is a solution to the e-NTRU problem.
(ii) If **v**^*∗*^ appears in the *H* query and is stored as ((*r*_*i*,0_, *r*_*i*,1_)_1≤*i*≤*l*_, *L*, *m*, *I*, *v*^*∗*^, *k*) in *list*_*H*_, then,
  (26) $$\begin{array}{c} v^{*}=H\left(\sum_{1\leq i\leq l}\left(r_{i,0}+r_{i,1}*h_{i}\right),L,m,I\right). \end{array}$$
When the function *H* collides, *S* aborts (Abort II). Otherwise, from (23) and (26), there is
  (27) $$\begin{array}{c} L^{*}=L,m^{*}=m,I^{*}=I,\\ \sum_{1\leq i\leq l}\left(z_{i,0}^{*}+z_{i,1}^{*}*h_{i}\right)-I^{*}v^{*}=\sum_{1\leq i\leq l}\left(r_{i,0}+r_{i,1}*h_{i}\right). \end{array}$$
*S* performs the following: when *i* ≠ *k*^*∗*^, let **z**_*i*,0_=**r**_*i*,0_ and **z**_*i*,1_=**r**_*i*,1_; when *i* = *k*^*∗*^, let *z*_*k*^*∗*^,0_=*r*_*k*^*∗*^,0_+*v*^*∗*^*I* and **z**_*k*^*∗*^,1_=**r**_*k*^*∗*^,1_. Then, we have
  (28) $$\begin{array}{c} v^{*}=H\left(\sum_{1\leq i\leq l}\left(r_{i,0}+r_{i,1}*h_{i}\right),L,m,I\right)\\ =H\left(\sum_{1\leq i\leq l,i\neq k^{*}}\left(r_{i,0}+r_{i,1}*h_{i}\right)+z_{k^{*},0}-v^{*}I+z_{k^{*},1}*h_{k^{*}},L,m,I\right)\\ =H\left(\left.\sum_{1\leq i\leq l}\left(r_{i,0}+r_{i,1}*h_{i}\right)\right)-v^{*}I,L,m,I\right). \end{array}$$
Given (23), (27), and (28), we have
  (29) $$\begin{array}{c} \sum_{1\leq i\leq l}\left(z_{i,0}-z_{i,0}^{*}\right)+\left(z_{i,1}-z_{i,1}^{*}\right)*h_{i}=0\mathrm{mod}q. \end{array}$$
Thus, the solution to the e-NTRU problem is [(**z**_*i*,0_^*∗*^ − **z**_*i*,0_),(**z**_*i*,0_^*∗*^ − **z**_*i*,1_)]_1≤*i*≤*l*_.

*Probability Analysis*. The challenger *S* fails when Aborts I and II occur. The probability of *H* colliding is 1/2^*n*^. Assume *A* can successfully forge the signature with probability *ξ*, then the probability of *S* solving the e-NTRU problem is *ξ* − 1/2^*n*^ × 2=*ξ* − 1/2^*n*−1^. This completes the proof.

**Theorem 4 (unconditional anonymity).**

The proposed scheme satisfies unconditional anonymity.

**Proof**

The anonymity proof of the signature is completed by the following game between adversary *A* and challenger *S*. If the signature distributions of *l* different members in the ring are computationally indistinguishable to adversary *A*, then this scheme satisfies anonymity.

 
*Query Phase*: *A* is allowed to access *JO*, and *S* responds as follows:
 
*JO*: *A* inputs an index *j* to query. *S* runs the algorithm KeyGen(*PP*) to generate the public key *pk*_*j*_=*h*_*j*_ and returns it to *A.*
 
*Challenge Phase*: *A* inputs a public key list *L*={*h*_*i*_}_1≤*i*≤*l*_, and a message *m*^*∗*^ ∈ {0,1}^*∗*^. *S* randomly chooses *b* ∈ {1, ⋯, *l*}, then runs Sign(*PP*, *L*, *m*^*∗*^, *sk*_*b*_) to generate the signature *σ*(*m*^*∗*^)=Sign(*PP*, *L*, *m*^*∗*^, *sk*_*b*_) and gives it *A*, where *sk*_*b*_ is the private key corresponding to index *b*.
 
*Guess Phase*: *A* gives a value *b*′∈{1, ⋯, *l*} as a guess for *b*.
 
*Analysis*. Suppose *A* is an adversary with unlimited computing power. Next, we will show the advantage *Adv*_*A*_^*Anon*^ of *A* in winning the anonymous game is negligible. We need to prove that the distributions of signatures generated with the private keys of different users are computationally indistinguishable.

First, even *A* is an adversary with unlimited computing power, from the *JO* query, or from the challenger signature (which contains a linkability tag), *A* still cannot deduce the private key, as well as the corresponding index. That is because the randomness of the algorithms TrapGen and SamplePre makes each public key *h*_*b*_ correspond to multiple pairs (*s*_*b*,0_, *s*_*b*,1_), and which one is the actual private key of member *b* cannot be determined. Moreover, given a linking tag *I*=*s*_*b*,0_+*s*_*b*,1_*∗h*_*b*_, to know which member generated the linking tag *I*, it is no better than random guessing for the adversary. In addition, it should be noticed that the signature *σ*(*m*^*∗*^) is generated by using not only a private key (*s*_*b*,0_, *s*_*b*,1_) but also a set of random numbers. Lemma 3 guarantees that the distributions of (**z**_*b*,0_, **z**_*b*,1_) and (**z**_*i*,0_,  **z**_*i*,1_)_*i*≠*b*_ are indistinguishable, and the distribution of (**z**_*b*,0_, **z**_*b*,1_) is independent of (*s*_*b*,0_, *s*_*b*,1_). That is, in the view of the adversary, the signature *σ*(*m*^*∗*^) is independent of the index *b* of the actual signer. Hence, we can conclude that even an unbounded adversary cannot guess the index *b* with a probability greater than 1/*l*.

We can infer that when *A* is a normal adversary, that is, *A* has limited computing power and time, obviously it ccannot destroy the anonymity of the scheme. This completes the proof.

**Theorem 5 (linkability).**

Under the random oracle model, if the proposed scheme is unforgeable, then for any PPT adversary *A*, the proposed scheme is linkable.

**Proof**

We will show that if the proposed scheme satisfies unforgeability, then it will satisfy linkability. The linkability proof of the scheme is completed by the following game interaction between an adversary *A* and a challenger *S*.

(i) *S* generates the system public parameters *PP* and public and private keys (*pk*_*i*_, *sk*_*i*_)_1≤*i*≤*l*_, and then sends *PP* to *A*
(ii) *A* can access *JO*, *CO*, *SO*, and *HO*, and the process of accessing *JO*, *CO*, *SO*, and *HO* in the linkability game is the same as that in the unforgeability game
(iii) Suppose *A* outputs two signatures *σ*_1_(*m*_1_)=(*m*_1_, (*z*_*i*,0_, *z*_*i*,1_)_1≤*i*≤*l*_, *v*_1_, *I*_1_) and *σ*_2_(*m*_2_)=(*m*_2_, (**z**_*i*,0_′, **z**_*i*,1_′)_1≤*i*≤*l*_, **v**_2_, *I*_2_) under public key set *L*, which satisfy the following conditions:
  1. All public keys in *L* are outputs of *JO*
  2. For *i*=1,2, *Verify*(*PP*, *L*, *m*_*i*_, *σ*_*i*_(*m*_*i*_))=^″^1^″^ such that *σ*_*i*_(*m*_*i*_) is not an output of *SO*
  3. *A* accesses *CO* once at most

*Analysis*. Assume *A* can generate two signatures *σ*_1_(*m*_1_) and *σ*_2_(*m*_2_) with a nonnegligible probability *η* while holding only one private key *sk*_*k*_, and ^″^1^″^ ← Verify(*PP*, *L*, *m*_*i*_, *σ*_*i*_(*m*_*i*_)) for *i*=1,2. Given that the proposed LRS scheme is unforgeable, these two signatures can be validated by the *Verify* algorithm if and only if *A* honestly generates signatures *σ*_1_(*m*_1_) and *σ*_2_(*m*_2_) using his private key *sk*_*k*_. In other words, we have *I*_1_=*s*_*k*,0_+*s*_*k*,1_*∗h*_*k*_ and *I*_2_=*s*_*k*,0_+*s*_*k*,1_*∗h*_*k*_′. And since there is also only one public key corresponding to this private key, that is, *h*_*k*_=*h*_*k*_′, we have *I*_1_=*I*_2_. This indicates that the algorithm Link(*σ*_1_(*m*_1_), *σ*_2_(*m*_2_)) returns “Link“ when given two signatures *σ*_1_(*m*_1_) and *σ*_2_(*m*_2_). Hence, the advantage *Adv*_*A*_^*link*^ of *A* is negligible. This completes the proof.

## 6. Discussion

### 6.1. Parameter Selection

The security of the proposed scheme is based on the e-NTRU problem, which is reduced to the NTRU-SIS problem. The NTRU-SIS problem is to find two polynomials (*u*, *v*) ∈ *R*_*q*_^2^ that satisfies *u*+*v∗h*=0mod*q* and ‖*u*‖, ‖*v*‖ ≤ *β* in the NTRU lattice, which is in turn reduced to *γ*-Ideal-SVP problem. Similar to [34, 36], we use the “root Hermite factor *γ*” which measures the hardness of *γ*-Ideal-SVP problems to select the parameters.

If we look for a polynomial *v* in an *n*-dimensional lattice, which is greater than the *n*-th root of the determinant, then the associated *γ* is

(30) $$\begin{array}{c} \frac{\left\|v\right\|}{\det{\left(\Lambda \right)}^{1/n}}=\gamma^{n}. \end{array}$$

According to [37], if we look for a small-size polynomial *v* in the NTRU lattice, the associated *γ* is

(31) $$\begin{array}{c} \frac{\sqrt{n/\left(2\pi e\right)}\cdot \det{\left(\Lambda \right)}^{1/n}}{\left\|v\right\|}=0.4\gamma^{n}. \end{array}$$

From the results in [36, 38], if the value of *γ* is approximately 1.007, to find the polynomial is at least 80 bits hard. If the value of *γ* is less than 1.004, to find the polynomial is at least 192 bits hard.

The methods to attack the proposed scheme are mainly to attack the ring member's public key and the signature.

The public key of the member *i* is a polynomial *h*_*i*_=*g*_*i*_*∗f*_*i*_^−1^mod*q* ∈ *R*_*q*_. The attack on *h*_*i*_ is to find two nonzero small-size polynomial (*u*_*i*_, *v*_*i*_) ∈ *R*_*q*_^2^ that satisfies *u*_*i*_+*v*_*i*_*∗h*_*i*_=0mod*q*. By Lemma 1 we know, $\left\|\left(u_{i},v_{i}\right)\right\|\leq \sigma \sqrt{2n}$. So using (32) to calculate the value of *γ*, we have $\gamma ={\left(\sqrt{n}/1.368\right)}^{1/2n}$. When *n*=256, *γ* ≈ 1.0048, it is at least 80 bits hard to attack the ring member's public key, and when *n*=512, *γ* ≈ 1.0027, it is at least 192 bits hard to attack the ring member's public key.

The attack on the signature of the member *i* is to find a vector (**z**_*i*,0_, **z**_*i*,1_) passing the verification algorithm without member *i*′*s* private key. It can be seen from Lemma 3, $\left\|\left(z_{i,0},z_{i,1}\right)\right\|\leq s\sqrt{2n}$. Since $s=0.585/\pi \sqrt{q\;\ln\left(2+2/\eta \right)}$, where *η*=2^−*λ*^/2*n*, there is $s=1.4708\sqrt{q}$ for *n*=256 and $s=2.2089\sqrt{q}$ for *n*=512. So, computing the value of *γ* by (28), we have

(32) $$\begin{array}{c} \frac{\sigma \sqrt{2n}}{\sqrt{q}}=\gamma^{2n}⟹\left\{\begin{array}{cc} \gamma ={\left(2.080\sqrt{n}\right)}^{1/2n}, & n=256\\ \gamma ={\left(3.124\sqrt{n}\right)}^{1.2n} & n=512 \end{array}\right). \end{array}$$

When *n*=256, *γ* ≈ 1.0069, to attack the ring member's signature is at least 80 bits hard, and when *n*=512, *γ* ≈ 1.0041, to attack the ring member's signature is at least 192 bits hard. The recommended choice of the parameters is shown in Table 2.

### 6.2. Post-Quantum Security

The proposed scheme is based on the hard assumption over lattice which is generally recognized to provide anti-quantum security. The security proof of the proposed scheme is unlikely to be extended to the Quantum Random Oracle Model [39] (QROM): in the security proof (Theorems 3 and 5), we use the adaptive programming of random oracle (RO) *H*, and this proof technique is inherent in the structure to some extent.

We note that other schemes built on QROM, such as [40, 41], also use the form of RO programming (even if not adaptive). In addition, although Fiat–Shamir seems unlikely to be proved in QROM, to the best of our knowledge, there are no attacks on the protocols using these proof technologies, which stems from the use of RO.

## 7. Performance Analysis

In this section, the proposed LRS scheme is compared with the schemes [23, 24, 26, 27, 30] in terms of efficiency. We mainly compare these schemes in terms of elapsed time and storage space.

Comparison terms in Table 3 include signature generation cost, signature verification cost, unconditional anonymity, and difficult assumption. Comparison terms in Table 4 include public and private key, as well as signature size of each user. In Tables 3 and 4, *n* is the degree of polynomials, *q*=1mod2*n* is a large prime number, *l* represents the cardinality of the ring, and *k* and *v* are integers. The time cost for the discrete Gaussian sampling algorithm and the rejection sampling algorithm running once are represented by *T*_*SD*_ and *T*_*RS*_, respectively. In general, *T*_*SD*_ > *T*_*RS*_. The time cost for polynomial-polynomial multiplication is represented by *T*_*Mul*_, and *T*_*Mul*_ > *T*_*SD*_. The time overhead of hash, matrix-matrix addition, and polynomial-polynomial addition is ignored because these operations take less time. We mainly focus on time-consuming operations, such as matrix-matrix multiplication and polynomial-polynomial multiplication.

In terms of signature generation cost, the proposed scheme mainly uses the Gaussian sampling algorithm 2*l* times, the polynomial-polynomial multiplication *l* times, and the rejection sampling algorithm once, respectively. Hence, the signature generation cost is 2*nlT*_*SD*_+*nlT*_*Mu*l_+2*nT*_*RS*_. In terms of signature verification cost, since the proposed scheme primarily runs polynomial-polynomial multiplication *l* times, the signature generation cost is about *nlT*_*Mu*l_. From Table 3, due to *T*_*Mul*_ > *T*_*SD*_ > *T*_*RS*_, compared with the four schemes of [23, 24, 26, 30], the proposed scheme has higher signature generation and verification efficiency. The signature generation and verification time of the proposed scheme is linearly related to the number of ring members *l*, while that of the scheme of [27] has a logarithmic relationship with *l*. Therefore, when *l* is large, the signature generation and verification efficiency of the scheme of [27] is better than that of the proposed scheme. But when *l* is small, the proposed scheme is more efficient by the settings of relevant parameters. In addition, only Alberto Torres et al.'s scheme [24] and our scheme can achieve unconditional anonymity, while other four schemes only have computational anonymity. And the efficiency of signature generation and verification of our scheme is obviously higher than that of Torres et al.'s scheme.

In the proposed scheme, the public key of the member in the ring is a small polynomial *h*_*i*_ ∈ *R*_*q*_ generated by the trapdoor generation algorithm *TrapGen*, and the private key corresponds to two small polynomials in *R*_*q*_. Therefore, the public and private key lengths of the proposed scheme are *n*  log*q* and 2*n*  log*q*, respectively. As shown in Table 4, the public and private key lengths of [23, 24, 26, 27, 30] are (*kn*  log*q*, *n*  log*q*), (*n*  log*q*, *kn*  log*q*), (3*kn*  log*q*, *kn*  log*q*), (*kvn*  log*q*, *vn*  log*q*), and (*n*  log*q*, 9*n*  log*q*), respectively. Hence, in terms of public key size, the public key size of the proposed scheme is similar to that of [24, 30] and smaller than that of [23, 26, 27]. With respect to private key size, the private key size of the proposed scheme is larger than that of [23] and they are both smaller than that of [24, 26, 27, 30]. For signature size, the signature size of the scheme [27] has a logarithmic relationship with *l*, while that of the other five schemes including the proposed scheme has a linear relationship with *l*. But the growth rate of signature size of [23, 30] and the proposed scheme is obviously slower than that of [24, 26].

## 8. Implementation and Evaluation

We implemented and evaluated the proposed LRS scheme on a typical laptop configured with a Windows 8.1 system, an Intel(R) Core(TM) i5-4210U CPU@1.70 GHz processor, and a 4.00 GB running memory. We selected parameters to make the proposed scheme secure, and detailed parameter settings are given in Table 5. We ran the signature generation and verification algorithms for 1000 times. And at security level *λ*=80, the average running time of these algorithms of the five schemes under different numbers of ring members is shown in Table 6. It can be seen from Table 6 that the signature generation and verification of [24] take the longest time among the six schemes, while the signature generation and verification time of the proposed scheme is shorter than that of [23, 24, 26, 30]. Compared with [27], when *l* ≤ 256, the proposed scheme has higher signature efficiency, but when *l* ≥ 512, the signature efficiency of the proposed scheme needs to be improved. On average, compared with the other five schemes, the signature generation and verification time of the proposed scheme is reduced by about 56.61% and 65.18%, respectively. Especially compared with [24], which also has unconditional anonymity as ours, the signature generation and verification time of the proposed scheme is reduced by about 94.52% and 97.18%, respectively.

At security level *λ*=80, the comparison between the proposed scheme and the other five schemes on public/private key size and signature size under different numbers of ring members is shown in Table 7. As for the public key size, the public key size of the proposed scheme is equal to that of [24, 30] and smaller than that of [23, 26, 27]. With respect to private key size, the private key size of the proposed scheme is larger than that of [23] but is significantly smaller than that of [24, 26, 27, 30]. In the case of signature size, the signature size of the proposed scheme is larger than that of [23] but is significantly smaller than that of [24, 26, 30]. When *l* ≥ 64, the signature size of the scheme in [27] is shorter than that of the proposed scheme. However, the scheme of [27] only has computational anonymity, while the proposed scheme has unconditional anonymity. Especially compared with [24], the signature size of the proposed scheme is reduced by 58.03% on average.

In addition, in the above experiment, we only completed the proof-of-concept work and did not consider potential optimization algorithms, such as the polynomial-polynomial multiplication based on FFT.

## 9. Conclusions

Based on the e-NTRU problem, this study constructed a LRS scheme on NTRU lattice by combining preimage and rejection sampling techniques. Under the random oracle model, the security of our LRS scheme was analyzed in detail. The analysis results show that our scheme satisfies the requirements of correctness, unforgeability, and linkability based on the intractability of the e-NTRU problem in the random oracle model. In particular, our scheme can achieve unconditional anonymity. The efficiency of the proposed scheme was analyzed in detail, and the optional parameter settings of the proposed scheme that meet the security requirements are given. Finally, the proposed scheme and other five latest lattice-based LRS schemes are implemented, which shows that under the same security level, the proposed scheme has higher signature generation and verification efficiency as well as shorter signature size compared with other five LRS schemes.

## Figures and Tables

**Table 1:** Symbol description.

Notations	Explanation
*ℝ*	Set of real numbers
*ℤ*	Set of integers
*ℤ*_*q*_	Set of integers modulo *q*
*ℤ* ^*m*^	Set of *m*-dimensional column vectors over *ℤ*
*ℤ*_*q*_^*n*×*m*^	Set of matrices of *n* rows and *m* columns over *ℤ*_*q*_
*R*	Polynomial ring *ℤ*[*x*]/(*x*^*n*^+1)
*R*_*q*_	Polynomial ring *ℤ*_*q*_[*x*]/(*x*^*n*^+1)
**A**	Matrix
**x**	Vector
**x** ← *D*	Randomly choosing vector **x** from probability distribution *D*
‖**x**‖	Euclidean norm of vector **x**
*f* · *g*	Multiplication of polynomials
*negl*(*n*)	Negligible function about *n*
*g*(*n*)=*ω*(*f*(*n*))	*g*(*n*) > *f*(*n*)

**Table 2:** Parameter settings.

Parameter	Recommended choice	
*λ*	80	192
*γ*	1.0069	1.0040
*n*	256	512

**Table 3:** Comparison of time costs and difficult assumption.

Scheme	Signature cost	Verification cost	Unconditional anonymity	Difficult assumption
[23]	*nlT*_*SD*_+*kn*(2*l* − 1)*T*_*Mul*_+*nT*_*RS*_	2*knlT*_Mul_	No	MSIS, MLWE
[24]	*knlT*_*SD*_+*k*^2^*n*(2*l*+1)*T*_*M*ul_+*knT*_*RS*_	2*k*^2^*nlT*_Mul_	Yes	R-SIS
[26]	*knlT*_*SD*_+*kn*(2*l*+1)*T*_*Mul*_+*knT*_*RS*_	2*knlT*_*Mul*_	No	MSIS, MLWE
[27]	*vnT*_*SD*_+5*knT*_*Mul*_log*l*	2*knT*_*Mul*_log*l*	No	MSIS, MLWE
[30]	2*n*(*l*+1)*T*_*SD*_+2*n*(*l*+1)*T*_*Mu*l_	2*nlT*_Mul_	No	R-SIS,R-ISIS
Ours	2*nlT*_*SD*_+*nlT*_*Mu*l_+2*nT*_*RS*_	*nlT*_Mul_	Yes	e-NTRU

**Table 4:** Comparison of communication costs.

Scheme	Public key size (bits)	Private key size (bits)	Signature size (bits)
[23]	*kn* log*q*	*n* log*q*	*O*(*n* · *l*)
[24]	*n* log*q*	*kn* log*q*	*O*(*kn* · *l*)
[26]	3*kn* log*q*	*kn* log*q*	*O*(*kn* · *l*)
[27]	*kvn* log*q*	*vn* log*q*	*O*(*n* · log*l*)
[30]	*n* log*q*	9*n* log*q*	*O*(*n* · *l*)
Ours	*n* log*q*	2*n* log*q*	*O*(*n* · *l*)

**Table 5:** Parameter settings for our scheme.

Parameter	*n*	*k*	*v*	*q*	Security level
Recommended choice	256	5	4	2^32^	80 bits

**Table 6:** Comparison of time costs (ms) at security level *λ*=80.

Scheme	Signature time								Verification time							
	*l=*1	*l* = 8	*l* = 64	*l* = 128	*l* = 256	*l* = 512	*l* = 1024	*l* = 2048	*l* = 1	*l* = 8	*l* = 64	*l* = 128	*l* = 256	*l* = 512	*l* = 1024	*l* = 2048
[23]	3.42	33.44	218.09	421.31	817.84	1532.27	2953.14	5666.39	5.73	35.46	200.10	379.92	750.35	1410.44	2699.05	5189.23
[24]	32.87	154.36	1019.57	1906.38	3693.68	7084.92	14018.56	27925.31	24.32	133.12	950.27	1769.47	3440.64	6619.14	13120.31	23855.36
[26]	9.25	48.87	282.71	525.43	1002.42	1890.29	3659.71	7106.84	5.63	36.86	201.19	389.94	753.65	1428.68	2752.51	5295.31
[27]	34.97	101.14	202.98	239.61	281.36	330.24	381.64	447.12	14.02	41.56	83.32	100.15	121.06	150.38	176.86	237.63
[30]	3.78	14.88	82.87	151.91	265.57	514.81	986.62	1898.85	1.23	7.78	49.81	94.37	162.53	316.15	622.34	1172.31
Ours	1.71	10.36	59.56	109.04	199.47	364.63	688.64	1367.06	0.73	4.63	29.13	49.81	94.37	162.53	316.15	622.34

**Table 7:** Comparison of storage overhead (KB) at security level *λ*=80.

Scheme	[23]	[24]	[26]	[27]	[30]	Ours
Size of public key	5.45	1.09	16.35	31.80	1.09	1.09
Size of private key	1.09	5.45	5.45	6.36	9.81	2.18
Signature size for *l* *=* 1	6.54	6.54	7.63	33.39	4.36	3.27
Signature size for *l* *=* 8	14.17	44.69	45.78	36.57	27.25	18.53
Signature size for *l* *=* 64	75.21	349.89	350.98	41.34	210.37	140.61
Signature size for *l* *=* 128	144.97	698.69	699.78	42.93	419.65	280.13
Signature size for *l* *=* 256	284.49	1396.29	1397.38	44.52	838.21	559.17
Signature size for *l* *=* 512	563.53	2791.49	2792.58	46.11	1675.33	1117.25

## Data Availability

The data that support of our findings are available at https://github.com/wang-0218/ring-signature.
